# Prevalence of diarrheal disease and associated factors among under-five children in flood-prone settlements of Northwest Ethiopia: A cross-sectional community-based study

**DOI:** 10.3389/fped.2023.1056129

**Published:** 2023-01-23

**Authors:** Tsegaye Adane Birhan, Bikes Destaw Bitew, Henok Dagne, Dagnachew Eyachew Amare, Jember Azanaw, Mengesha Genet, Garedew Tadege Engdaw, Amensisa Hailu Tesfaye, Getasew Yirdaw, Tadele Maru

**Affiliations:** ^1^ Department of Environmental & Occupational Health & Safety, College of Medicine and Health Sciences, University of Gondar, Gondar, Ethiopia.; ^2^ Department of Environmental Health, College of Health Sciences, Debre Markos University, Debre Markos, Ethiopia; ^3^ Department of Environmental Health, Teda College of Health Sciences, Gondar, Ethiopia.

**Keywords:** diarrheal disease, flood-prone, under-five children, drinking water, Ethiopia

## Abstract

**Background:**

Diarrheal illnesses are a long-standing public health problem in developing countries due to numerous sanitation issues and a lack of safe drinking water. Floods exacerbate public health issues by spreading water-borne infectious diseases such as diarrhea through the destruction of sanitation facilities and contamination of drinking water. There has been a shortage of studies regarding the magnitude of diarrheal disease in flood-prone areas. Therefore, this research aimed to evaluate the prevalence of diarrheal disease and its predictors among under-five children living in flood-prone localities in the south Gondar zone of Northwest Ethiopia.

**Method:**

A community-based cross-sectional research was carried out in flood-prone villages of the Fogera and Libokemkem districts from March 17 to March 30, 2021. Purposive and systematic sampling techniques were used to select six kebeles and 717 study units, respectively. Structured and pretested questionnaires were used to collect the data. A multivariable analysis was performed to determine the predictors of diarrheal disease, with *P*-value <0.05 used as the cut-off point to declare the association.

**Result:**

The prevalence of a diarrheal disease among under-five children was 29.0%. The regular cleaning of the compound [AOR: 2.13; 95% CI (1.25, 3.62)], source of drinking water [AOR: 2.36; 95% CI: (1.26, 4.41)], animal access to water storage site [AOR: 3.04; 95% CI: (1.76, 5.24)], vector around food storage sites [AOR: 9.13; 95% CI: (4.06, 20.52)], use of leftover food [AOR: 4.31; 95% CI: (2.64, 7.04)], and fecal contamination of water [AOR: 12.56; 95% CI: (6.83, 23.20)] remained to have a significant association with diarrheal diseases.

**Conclusion:**

The present study found that the prevalence of the diarrheal disease among under-five children was high. Routine compound cleaning, the source of drinking water, animal access to a water storage site, vectors near food storage sites, consumption of leftover food, and fecal contamination of water were significant predictors of diarrheal disease. Therefore, it is advised to provide improved water sources, encourage routine cleaning of the living area, and offer health education about water, hygiene, and sanitation.

## Background

Diarrheal diseases are continued public health problems in developing countries as a result of rapid urbanization and its accompanying sanitation issues, along with a shortage of safe water to drink. Poor water and sanitation are responsible for over 94 percent of the four billion cases of diarrhea that occur each year around the world (1, 2). Furthermore, diarrhea kills approximately two million people per year, accounting for 4% of global mortality. In addition, diarrhea kills 1.3 million children per year (3). According to studies, children under the age of five years in underdeveloped nations experience three episodes of diarrhea each year on average (4, 5). It is also recognized that the disease mainly affects poverty-stricken populations (5).

The problem of diarrhea among under-five children is distributed unevenly across Africa. Diarrhea is particularly common among children in developing countries (6). Over half of all diarrhea-related deaths among African children are thought to arise in seven percent of the lower-level governmental entities of the continent (6). In 2016, over 75% of disease categories recorded in the Global Health Observatory showed serious environmental ties, and environmental conditions were responsible for more than 1.6 million under-five child fatalities (7). Diarrheal diseases account for 22% of the total disease burden in the environment, whereas parasitic and vector-borne diseases account for 12% (8).

According to retrospective analysis, 2.4 billion people use sanitation facilities that do not meet healthcare needs standards (9). Despite this, the frequency is extremely high, particularly in flood-prone areas. Diarrhea is more prevalent in developing nations than in industrialized nations. This is caused by several factors, including a lack of safe drinking water, poor knowledge about sanitation and hygiene, contamination of water with fecal coliforms, the use of left-over food, and low nutritional and public health status. One billion people lack access to safe drinking water, while about 2.5 billion people lack proper sanitary facilities (10).

According to recent national figures, the prevalence of diarrhea in children for the first two weeks was around 13% (11). Furthermore, according to a few local studies, the magnitude of diarrhea among under-five children in various parts of the country ranges from 18 to 31% (12–14).

Floods exacerbate public health issues by spreading water-borne infectious diseases such as diarrhea (15). However, the study site is one where certain villages have been regularly flooded due to its plain landscape. It is an appropriate resource for identifying and learning about settlements that are constantly flooded, as well as the prevalence of diarrheal disease. Despite the existence of this evidence, there hasn’t been enough research conducted in the country to get current data on the disease in flood-prone areas and to help decision-makers prioritize strategies to solve the problem. Therefore, the objective of this research was to examine the prevalence of diarrheal disease and its predictors among under-five children living in flood-prone localities in the south Gondar zone of Northwest Ethiopia.

## Materials and methods

### Study area, design, and period

**Figure 1:**
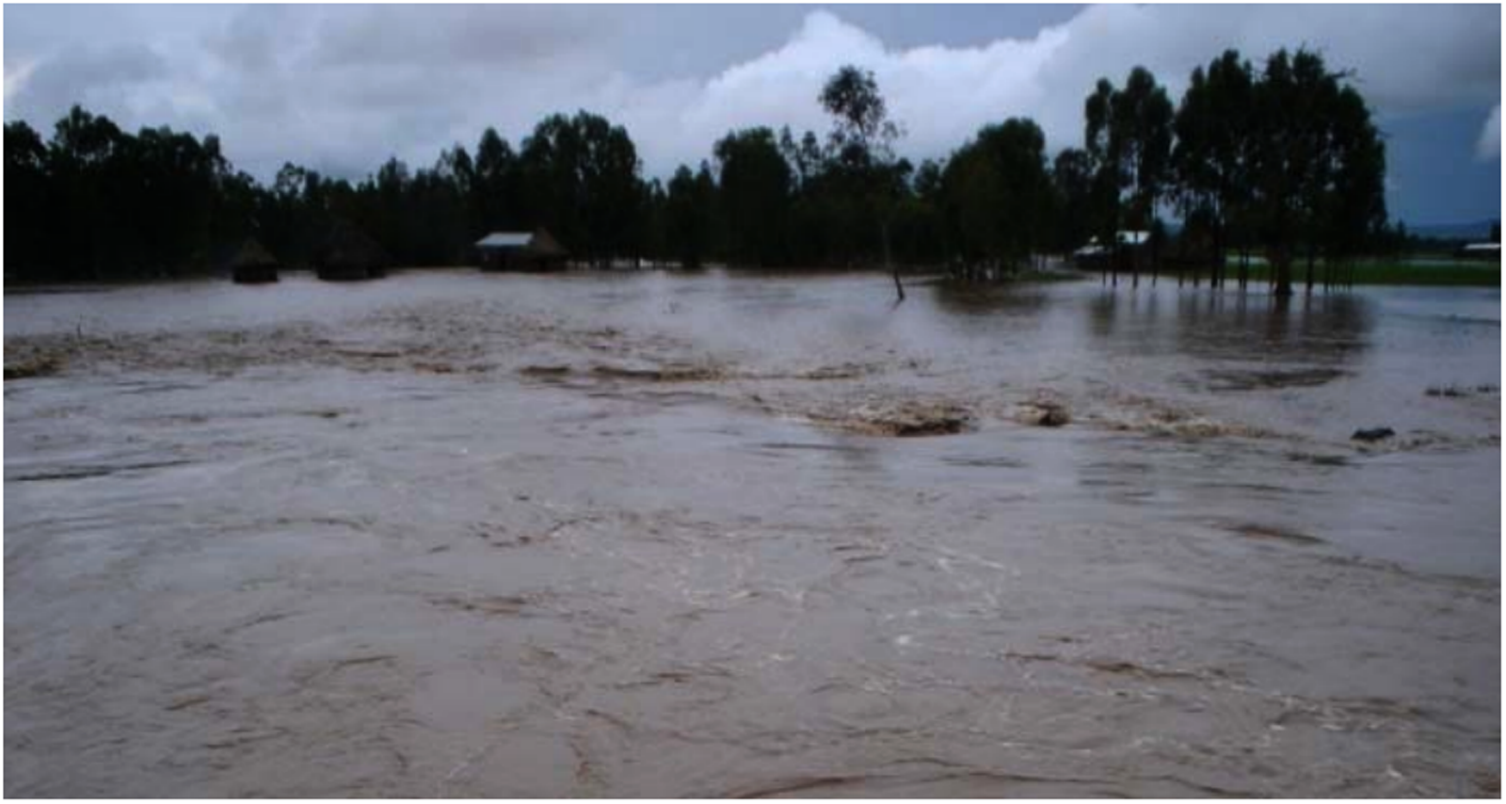
Flood case in the study area (in Ribb River Catchment area) (a Picture taken by *Woubet Gashaw Alemu in 2007* (*19*).

Community-based cross-sectional research was employed in flood-prone settings of Fogera and Libo Kemkem districts from March 17 to March 30; 2021. The districts of Fogera and Libo Kemkem are situated in Northwest Ethiopia, in the Amhara Regional State, in the watershed of the Ribb River, and close to Lake Tana, at an altitude of 2,000 meters above sea level. Details of the location of the study area and the population size of each district have been mentioned in our previous work (16). These flood-prone settlements repeatedly suffer from floods due to the overflow of the Ribb River (Figure 1). Currently, there is also flooding that may destroy sanitary facilities and water supply systems; that may also lead to poor water quality and inadequate sanitation and makes people more vulnerable to getting the infection. On September 15, 2020, a statement from the Amhara Media Corporation (Regional media) stated that the current flooding had impacted and dislocated around 2,439 and 1,750 households from Fogera and Libo Kemkem districts, respectively.

### Source and study population

The source and study population have been defined in the previously published work (16).

### Eligibility criteria

Households with under-five children with residents who lived for over six months and were present during data collection were included. While those households with mothers/caregivers who were not willing to participate or unable to respond due to serious illness were excluded. In cases where more than one under-five children are found in the household, the child who had recent instances of diarrhea was deemed qualified.

### Sample size determination

The following presumptions are made while calculating the sample size using a single population proportion formula: Diarrhea affected 30.5% of under-five children 30.5% (18), with a 5% margin of error (d) and 95% confidence interval.


$$n=\frac{{\left(Za_{/2}\right)}^{2}*P\left(1-P\right)}{d^{2}}\;n=\frac{{\left(1.96\right)}^{2}*\;0.35\left(1-0.35\right)}{{\left(0.05\right)}^{2}}\;=326$$

The overall sample size was 717 when the non-response rate of as10% and the design effect of 2 were taken into account.

### Sampling technique and procedure

Three kebeles in each of the two districts (Fogera and Libo Kemkem weredas) are selected in the rule of thumb principle based on their proximity to Ribb River to evaluate the magnitude of diarrheal disease and its predictors among under-five children in flood-prone localities. Based on their vulnerability to the frequent flooding and overflow of the river, the six kebeles (three from each district) as namely mentioned in our previously published work (16), were chosen for recruitment. Then, samples were recruited through proportional allocation followed by systematic sampling.

### Study variables

The dependent variable is the prevalence of diarrheal disease, whereas socio-demographic information of children and mothers: including sex, age, education attainment, occupational status, income, and religion; environmental factors: housing condition, types of water source, distance from a water source, type of water storage container, latrine accessibility and use, waste management practices; behavioral factors: children and mother's hygiene and hand washing practice, food hygiene and safety were considered as predictor variables.

### Data collection tool and procedure

The socio-demographic, water, hygiene, sanitation, and diarrheal disease information were gathered through a pretested structured questionnaire established by reviewing the literature (19–22). The questionnaire contains four main parts. Part-I contains the demographic and socio-economic condition of the family; and comprises a list of 18 items. Part-II also contains environmental and sanitation conditions including housing conditions, water quality, and safety measures, waste management practices including human excreta; and it also comprises 33 items, whereas Part-III contains the hygiene and behavioral conditions including food hygiene and safety, personal hygiene of the child and mothers/caregivers; and comprises 28 items. Then Part-IV also contains demographic, and diarrheal information about the child; which comprises 15 items. Some items in the questionnaire are checklists and can be collected through observation, whereas some items needed to be collected through face-to-face interviews with mothers/caregivers. Checklists were employed to monitor living conditions and housing situations. Six environmental health experts have examined the questionnaire's content and construct validity. Questionnaires were made to the mothers or caregivers of the child.

The bacteriological contamination [fecal coliform (FC)] of household drinking water was examined with the membrane filtration technique following the standard procedures (23). The details of water quality analysis procedures were explained in our previously published work (16).

### Operational definition

Improved water sources, unimproved water sources, and hand-washing practices at critical times have been defined in our previously published work (16).

**Diarrhea:** children who, according to their mother or other caregivers, have more than two loose or watery stools in 24 h.

**Acute diarrhea:** is a diarrheal episode that lasts fewer than 14 days.

**Fecal contamination of drinking water:** While the CFU/100 ml of water was greater than zero, they were categorized as “Yes”; while it was zero, then it was categorized as “No”.

### Data quality control

Three days of training on the objectives, procedures and strategies of data collection were given to data collectors and supervisors. To ensure its validity, the questionnaire was translated into the local tongue, Amharic, and then back again. Furthermore, it was pretested in a nearby district that was not used for the actual data collection. Moreover, there was strict supervision throughout the data collection process.

### Data analysis

Data were coded manually and then entered into EpiInfo^™^ 7, and exported to SPSS version 25 software for analysis. Descriptive statistics were performed to describe the study population with dependent and independent variables. Variables that appear to be associated (*p* < 0.2) in the bivariate binary logistic regression analysis are considered in the multivariable analysis. Variables scored *p* < 0.05 were deemed statistically substantial predictor variables. The Hosmer Lemeshow test was also used to gauge the model's fitness.

## Results

### Socio-demographic, environmental, and behavioral characteristics of respondents

The response rate was 94.1%. The socio-demographic characteristics, drinking water storage and handling practices, housing conditions, sanitation, and behavioral characteristics of the respondents have been shown in our previous published work (16).

### Prevalence of diarrheal disease

The overall prevalence of diarrhea among under-five children in the study setting was 29.0% with 95% CI: (25.5–32.6%).

### Factors associated with diarrheal disease

After governing for confounding variables in the multivariable binary logistic regression analysis, regular cleaning of the compound, source of drinking water, animal access to the water storage site, vector around food storage sites, use of leftover food, and fecal contamination of water remained to be significant predictors of diarrheal diseases.

Diarrhea was two times more probable in under-fives from households that neglected to regularly clean their complex than it was for those that did [AOR: 2.13; 95% CI (1.25, 3.62)]. The occurrences of diarrheal disease were significantly associated with the household's drinking water source. Households that used unimproved water sources increase the likelihood of childhood diarrhea by 2.36 times compared to households that used improved water source counterparts [AOR: 2.36; 95% CI: (1.26, 4.41)]. Compared to children whose homes did not have access to animals at water storage sites, the risk of acquiring under-five diarrheal illnesses was three times higher in families having access to animals at water storage sites [AOR: 3.04; 95% CI: (1.76, 5.24)]. Moreover, the probabilities of developing diarrhea were 9 times among children with a household's food storage site prone to vectors compared to children from households the food storage site is free from vectors [AOR: 9.13; 95% CI: (4.06, 20.52)]. Under five children who lived in families that had to use leftover food had an approximately 4.31-times higher chance of acquiring diarrheal disease compared to households that did not [AOR: 4.31; 95% CI: (2.64, 7.04)]. On top, the likelihoods of procuring the diarrheal disease were 12.56 times higher among under-five children from families who use faecally contaminated water compared with under-five children whose families utilized free from faecally contaminated water [AOR: 12.56; 95% CI: (6.83, 23.20)] (Table 1).

**Table 1:** Multivariable analysis of predictors for diarrhea in children under five living in flood-prone areas in Fogera and Libokemkem districts, Northwest Ethiopia, 2021 (*n* = 675).

Variables		Diarrheal diseased		COR (95% C.I)	AOR (95% C.I)
		Yes (%)	No (%)		
Regular cleaning of the compound	Yes	108 (16.00%)	324 (48.00%)	1.00	1.00
	No	88 (13.04%)	155 (22.96%)	1.70 (1.21, 2.39)	2.13 (1.25, 3.62)*
Condition of water storage	Covered	108 (16.00%)	288 (42.66%)	1.00	1.00
	Not covered	88 (13.04%)	191 (28.30%)	1.23 (0.88, 1.72)	1.01 (0.57, 1.81)
Source of drinking water	Improved	90 (13.33%)	234 (34.67%)	1.00	1.00
	Unimproved	106 (15.70%)	245 (36.30%)	1.13 (0.81, 1.57)	2.36 (1.26, 4.41)*
Number of water sources	One	27 (4.00%)	99 (14.66%)	1.00	1.00
	More than one	169 (25.04%)	380 (56.30%)	1.63 (1.03, 2.59)	1.63 (0.91, 2.92)
Animal access to the water storage site	Yes	63 (9.33%)	81 (12.00%)	2.33 (1.59, 3.41)	3.04 (1.76, 5.24)*
	No	133 (19.71%)	398 (58.96%)	1.00	1.00
Vector around food storage sites	Yes	187 (27.70%)	353 (52.30%)	7.42 (3.69, 14.92)	9.13 (4.06, 20.52)*
	No	9 (1.33%)	126 (18.67%)	1.00	1.00
Use leftover food	Yes	91 (13.48%)	80 (11.85%)	4.32 (2.99, 6.25)	4.31 (2.64, 7.04)*
	No	105 (15.56%)	399 (59.11%)	1.00	1.00
Fecal contamination of water	Positive	179 (26.52%)	240 (35.55%)	11.20 (6.51, 19.26)	12.56 (6.83, 23.20)*
	Negative	17 (2.52%)	239 (35.41%)	1.00	1.00

* Significant at *P*-value <0.05.

## Discussion

This community-based cross-sectional study was conducted to examine the prevalence of and predictors of diarrhea among under-five children in flood-prone settlements of Fogera and Libokemkem districts. In this study, the prevalence of diarrhea was 29.0%, with 95% CI: (25.5–32.6). Regular compound cleaning, source of drinking water, animal access to the water storage site, vectors around food storage sites, use of leftover food, and fecal contamination of water were significant predictors of diarrhea. The results of this investigation were higher than the other studies conducted in different parts of the country.

The 29.0% prevalence of this study is comparable with studies conducted in Yemen at 29.07% (24). This finding is also in line with previous research carried out in Uganda 29.1% (25), Arba-Minch District, 30.5%, Nekemte Town, 28.9% (26), Wonago District, 30.9% (27), and 28.4% in Harena Buluk Woreda (28). This similarity in the prevalence of diarrhea may be due to a similar status of the study area concerning its socioeconomic status and the level of practice of water, sanitation, and hygiene.

The prevalence of diarrhea from the present study is much higher than that reported in the Ethiopian DHS report of 12.0% (29). This is because the study is being conducted in flood-prone areas where flooding plays a significant role in exacerbating public health issues such as the spread of water-related communicable diseases such as diarrhea. Additionally, it is substantially higher than what was discovered in various regions of Ethiopia, such as in North Gondar Zone 23.2% (30), in Mecha District 22.1% (12), in Wolaita Sodo Town 11% (31), in Kersa District 22.5% (14), and Jabithennan District 21.5% (32). This could be because the area's being highly prone to flooding, and this condition is related to the lower establishment of services of water, sanitation, and hygiene, which in turn increases the prevalence of diarrhea in the study setting. The discrepancy could be the consequence of neglecting the execution of health extension programs by considering the constructed water, sanitation, and hygiene facilities are destroyed by floods (especially in the summer season).

From the current finding, the prevalence of diarrheal disease was associated with the practice of cleaning compounds regularly. Children from households that do not clean their compounds in a regular pattern or not at all were 2.13 times more probable to acquire the diarrheal disease than those from families that clean their compounds in a regular pattern. This finding was supported by previous findings from Addis Ababa (33), Rwanda (34), and Bangladesh (35). This could be the reason that if the compound is full of garbage, it creates a favorable environment for the breeding of numerous pests that may transmit diarrhea pathogens from the waste to food items.

Children in families receiving water from unimproved sources had a 2.36 times higher chance of getting diarrhea than children in households receiving water from improved sources. This may be linked with exposure to several microorganisms from polluted water may result in diarrhea illness. This finding was also consistent with multiple findings discovered from Amhara Region (36), Jigjiga District (37), Derashe District (38), Sheka Zone (39), Indonesia (40), and Uganda (25), however, it is inconsistent with findings reported from Farta Wereda (41), Debre Berhan Town (40), and Benishangul Gumuz (42). This variation may be because of the use of different water treatment practices in different localities.

This study also demonstrates a positive connection between households with cattle living in the same house and having access for animals to water storage areas with the prevalence of the diarrheal disease. Children from families without access to animals for water storage had a diarrhea risk that was more than three times lower than that of children from households with such access. This study is supported by studies conducted in Debre Berhan (43) and Harena Buluk Woreda, Oromia Region (28). However, it is in contrast with a study conducted in Tigray (44). This discrepancy may be brought about by the fact that putting animals in the same home is a prevalent practice in the study site, where flooding frequently affects the dwellings of cattle, which will live in the same home as inhabitants. This could potentially be a source of contamination.

This study also discovered a statistical association between feeding leftover meals to children and their diarrhea. Children whose mothers fed leftover food were 4.3 times more probable to acquire diarrhea than children from families that did not feed leftover food. It was also in line with previous findings from Hadaleala District (33), Afar Region, Northeast Ethiopia (45), Pader District, Northern Uganda (25), and Southern Nepal (46). Food that has been stored leftovers may be more likely to be contaminated by several germs that cause diarrhea. As a result, cooking food for children properly and serving it to them right away after cooking may lessen the chance of additional food contamination and prevent diarrhea.

We also discovered that diarrhea was substantially linked with fly and other vector activity at food storage sites. This finding is consistent with research from Addis Ababa (33). The lack of standards of cleanliness of the restrooms, the dumping of trash nearby the toilet facilities and within the residential enclosure, and the disposal of household sewage in these areas all seem to contribute to the presence of flies and other vectors in the surrounding food storage sites.

In the present study, children who used water that had fecal contamination had a 12.5 times higher chance of getting diarrhea than those who did not. This outcome is in line with findings from Nigeria (47). This is because the surroundings and the food that children eat can become contaminated by microorganisms in feces that are dumped in areas close to the house, which can cause diarrheal illness (48, 49).

### Strengths and limitations of the study

This study is the first to be carried out in flood-affected areas of the country after a flood season, and it may provide information to governmental and non-governmental organizations that deal with emergencies and disaster relief.

Since the study is cross-sectional, causation is not possible. There may be a social desirability bias and recall bias that may lead to incorrect reports. Due to limited resources, the study did not include a control group (households situated distant from the river). Even though we provided health education following data collection, we did not assess the change in handwashing practice, which is a critical issue during the COVID-19 pandemic. This study was also likewise conducted during the dry season and lacked seasonal adjustment.

## Conclusions

The prevalence of diarrheal disease among under-five children in the study setting was discovered to be high [29.0% with 95% CI: (25.5–32.6%)]. The regular cleaning of the compound, source of drinking water, animal access to the water storage site, vector around food storage sites, use of leftover food, and fecal contamination of water remained to have a significant association with under-five diarrheal diseases. Therefore, developing improved water sources, promoting regular cleaning of the living compound, and providing health education regarding water, hygiene and sanitation are recommended.

## Data Availability

The raw data supporting the conclusions of this article will be made available by the authors, without undue reservation.
